# Explanations for use of dietary- and muscle enhancing dietary supplements among university students: a national cross-sectional study

**DOI:** 10.1186/s40795-022-00510-1

**Published:** 2022-02-24

**Authors:** Christine Sundgot-Borgen, Therese Fostervold Mathisen, Monica Klungland Torstveit, Jorunn Sundgot-Borgen

**Affiliations:** 1 Department of Sports Medicine, Norwegian School of Sport Sciences, Sognsveien 220, N-0806 Oslo, Norway; 2 Regional Department for Eating Disorders, Division of Mental Health and Addiction, Oslo University Hospital, Postbox 4956, Nydalen, 0424 Oslo, Norway; 3 Faculty of Health, Welfare and Organization, Østfold University College, PO 700, 1757 Halden, Norway; 4 Faculty of Health and Sport Sciences, University of Agder, Postbox 422, 4604 Kristiansand, Norway

**Keywords:** Dietary supplements, Muscle enhancing supplements, Students, Body idealization, Internalization, Exercise science

## Abstract

**Background:**

Use of dietary supplements (DS) and muscle enhancing dietary supplements (MEDS) is frequent among students despite the lack of evidence of effects and health risks related to consumption. We need to increase our understanding of students’ motivation, examine potential gender differences, and explore explanatory factors, to address preventive measures related to use. Therefore, this study aimed to explore the frequency of, and the reasons for, DS use among university students, as well as explanatory factors for use of MEDS.

**Method:**

Male and female students from nine Norwegian universities participated in this cross-sectional study. Participants responded to questions about demographics, DS and MEDS use, internalization of body ideals, physical activity- and exercise level, motives for exercise, and exercise context. Independent t-test, Chi-square test, Pearson’s correlation, and logistic regression were used to investigate between group differences, associations, and explanatory factors for use, respectively. *P*-values ≤ 0.05 were defined as significant.

**Results:**

A total of 1001 males (34%) and females, with a mean(sd) age of 24.21(4.76) years, participated. The frequency of DS use was 42% and 40% (*p* = .414) in males and females, respectively, in which more males than females used DS to improve physical or mental performance (*p* =  < .001), increase muscle mass (*p* =  < .001) and weight for health (*p* = .014), and improve appearance (*p* =  < .001). In males and females, respectively, 25% and 10% used MEDS (*p* =  < .001). In males, being a fitness center member (OR:3.80), exercising to improve muscle mass (OR:1.96), and a higher physical activity level (OR:1.09) positively explained MEDS use, while exercising to increase endurance (OR:0.49) and being an exercise science student (OR:0.47) negatively explained MEDS use. In females, internalizing the athletic body ideal (OR:1.78) and exercising to improve muscle mass (OR:1.74) positively explained MEDS use.

**Conclusions:**

Our main findings indicate that a surprisingly high percentage of male and female Norwegian students use DS and MEDS, and that reasons for use, and variables explaining the variability in frequency of use, differ between genders. Longitudinal studies to investigate direction of associations are needed in future research.

**Supplementary Information:**

The online version contains supplementary material available at 10.1186/s40795-022-00510-1.

## Background

### Dietary supplement use among university students

Dietary supplements (DS) are generally defined as products which are orally consumed and aim to supplement one’s diet [1]. Studies show that higher education level is associated with more DS use compared to lower education level [2–4]. In fact, 43–80% of male and female American, Australian, and Canadian university students report use of DS, with use of vitamins/minerals, muscle enhancing dietary supplements (MEDS) (such as protein, creatine, amino acids), weight loss supplements, and herbs, being the most commonly reported [2, 5–9]. The most frequently reported reason for use is related to health promotion, followed by providing more energy, enhancing sport performance, and appearance improvement [2, 5, 9–11]. DS use among university students is associated with higher levels of physical activity, and lower body mass index (BMI) [5, 6, 9, 12, 13], and most studies report no gender difference in frequency of use [5, 6, 11, 13, 14].

The high frequency of DS use among students exists despite the scarce evidence on the efficacy and acute and long-term health effects from using such supplements [15]. This is a concern as public health guidelines do not include the use of DS, and use might therefore potentially increase the risk of an intake above the safety limits of various nutrients [16]. MEDS especially, have gone through poor quality control and have been found harmful due to contamination of illegal substances [17, 18]. Use of MEDS is also worrying because it is related to body image issues [19], which has been related to increased stress level and reduced quality of life in youth [20] and to reduced mental health in university students [21].

### The athletic body ideal and muscle enhancing dietary supplements

Unfortunately, especially within the marketing of MEDS, there is a strong idealization of extreme athletic bodies which facilitates internalization of these unhealthy and unrealistic ideals. Internalization of this specific ideal has been found to increase upward appearance comparison and body dissatisfaction in undergraduate males [22] and females [23] and might strengthen the perceived need to use supplements to obtain the internalized body type. In fact, studies on adolescents show that frequent users of MEDS are characterized by high scores on internalization of the athletic body ideal [24–26].

Parallel to the increased focus on the athletic body as a health ideal, the prevalence of older adolescents [27] and young adults [28] being a member at a fitness center has exploded. The fitness center environment is associated with pressure to comply with the athletic body ideal [21, 29, 30]. Also, fitness centers sell MEDS and are believed to stimulate use among members [31]. In support of this, studies have found that Norwegian adolescents [24], and American and Brazilian adults [31, 32], who were members at a fitness center, are frequent users.

Studies have also found that male and female students representing health related study programs report less use of DS in general compared to students in non-health related programs [7, 9, 33], while others find the opposite [10, 11, 13, 34]. However, previous studies have not focused on the use of MEDS, and students attending health focused exercise science study programs have not been included into studies comparing the influence of study program on DS use. Due to their health-, physical exercise-, and performance focused educational background, one might argue that exercise science students’ attitudes and behaviors related to MEDS would differ from those of other health-and non-health related-students.

Due to the potential physical and mental health risk of using DS in general, continuous tracking to evaluate trends in DS use among students, seems important. Studies on Nordic students are lacking, and further investigation of gender differences is needed. Finally, to our knowledge, no studies have investigated whether internalization of the athletic body ideal, being a fitness center member, or being an exercise science student or not, might be explanatory factors for the use of MEDS among university students.

Therefore, this study aimed to explore 1) the frequency of, and 2) the reasons for DS use among male and female university students. We also aimed to 3) explore the frequency of and identify variables explaining the variability in frequency of MEDS use, specifically evaluating internalization of body ideals, exercise context (i.e., participating in organized sports or being a fitness center member), study program (being an exercise science student or not), physical activity and exercise level, and motives for exercise, in both male and female university students.

## Method

### Study design

This study is a part of a large cross-sectional study where the overall aim was to explore the level and associations of body appreciation, body appearance pressure, body image related mental health constructs, physical activity and exercise level, and DS use in Norwegian university students [21]. Data on demographics, different aspects of DS use, physical activity and exercise, and internalization, are included in this current study.

### Participants

To achieve good representability of both exercise science, and non-exercise science students in Norway, nine universities from large cities, representing higher education campuses in all cardinal areas in Norway, offering exercise science and/or non-health related study programs, were contacted and asked to participate in the study. Eligibility criteria were being male and female students who understood Norwegian writing. Participants represented students enrolled in a bachelor’s or master’s degree in exercise sciences, teaching, engineering, or business and administration. Students were split into “male” (*n* = 335) and “female” (*n* = 666) to investigate gender differences, and either “exercise science students” (*n* = 517) or “other students” (*n* = 476), to investigate explanatory factors for MEDS use.

### Data collection

After consent from the dean at each university, the contact person at each universities’ faculties representing 1) exercise science programs and 2) non-exercise science or non-health related study programs, were asked if their students could be contacted by the research team. Students were then informed about the study and asked to participate through their student e-mail and web-based learning management systems. Students were recruited during the period of January-June 2020 and responded once to an electronic questionnaire. Prior to answering the questionnaire, students were informed about the overall aim of the study, and that participation would take about 15 min.

### Demographics

Students self-reported age, gender, height and weight, scientific study program (recoded into being an exercise science student (1) or not (0)), as well as immigration status, where having two parents immigrated defined a student with immigration background.

### Supplementation

Use of DS and MEDS were the main outcome variables. Outcomes were measured with self-developed questions on current use of DS (yes = 1, no = 0) and type of DS used (vitamins, minerals, MEDS (protein, creatine, amino acids), fat-burners, dieting aids, herbal products, meal replacements, and sports supplements (e.g., sports drinks, pre-workout, energy boosters, bars)). Students were also asked whether they used any type of DS which they assumed or knew was illegal in Norway (e.g., DS containing growth-hormones, pro-hormones, fat-burners, diuretics, stimulants, or sedatives). We also measured reasons for use of DS and illegal DS, with several response options (to maintain or improve health, improve mental or physical performance, increase muscle mass, reduce fat mass, increase weight for health, reduce weight for health, increase and reduce weight for appearance reasons). Students were free to report more than one type of DS and reason for use. To provide a more logical presentation of correlations and analyses evaluating explanatory factors for use of MEDS, we merged protein, creatine, and amino acid supplementations to one new variable defined as MEDS. Comparably, dieting supplements and fat-burners were merged into the group “Dieting aids”.

### Physical activity and exercise

Physical activity was in the questionnaire defined as “all bodily movement that lead to an increase in body temperature, and light-heavy shortness of breath” [35]. Students rated, in hours and minutes, their level of physical activity during the last week. Students also reported number of exercise sessions during the last week, where exercise was defined as “a type of physical activity conducted to maintain or improve physical fitness (e.g., resistance training, cardio). Exercise is more planned than regular physical activity” [35]. Data on physical activity and exercise were treated as continuous data in the analyses.

### Motives for exercise

Through a self-developed questionnaire, students were asked about their motives for exercise and responded to each motive (to prevent illness, reduce body weight, maintain body weight, increase muscle mass, reduce bodyfat percentage, change body shape, increase muscle strength, increase endurance, to perform well in physical activities or sports, because it is fun, it is social, it promotes good mood, to increase energy, reduce bad conscience) on a Likert scale ranging from 1 (*highly disagree*) to 5 (*fully agree*).

### Exercise context

Students were asked whether they currently participated in any organized sports (yes = 1, no = 0) and whether they were members of a fitness center (yes = 1, no = 0). Students could therefore be categorized as members within both exercise contexts.

### Sociocultural attitudes towards appearance questionnaire 4R (SATAQ-4R)

To assess internalization of body ideals, we used the internalization sub-tests Thin/low body fat internalization, Athletic/muscular internalization, and General attractiveness internalization in the SATAQ-4R [36]. Participants responded to a 5-point Likert-scale ranging from 1 (*strongly disagree*) to 5 (*strongly agree*), where negatively worded items were reversed and a mean score for each sub-test was calculated. A higher score indicates a higher degree of internalization. We found a good model fit for the current sample, and an α of 0.79-0.87 and 0.81-0.89 for male and females, respectively. This is similar to reported α in college male and females [36].

### Statistical analyzes

Data were analysed with IBM SPSS version 26. After visually evaluating the data for normality, between group differences were analyzed using student independent t-test for numerical data and Chi-square test for categorical data. The association between variables was evaluated by Person’s correlation and Chi-square test for numerical and categorical data, respectively, conducted with bootstrapping with 1000 replications. Correlation coefficients in the areas 0.1, 0.3 and 0.5 were interpreted as weak, moderate, and strong effect sizes, respectively [37]. (Data on group difference between MEDS users and non-users, and correlational data can be found within Additional Table 1 and Additional Table 2, respectively). Direct logistic regression was performed to assess the impact of independent variables (SATAQ, exercise context, study program, BMI, physical activity level, number of exercise sessions, and motives for exercise) on the dependent dichotomous variable use of MEDS. Independent variables which significantly correlated with the dependent variable (*r* > 0.2), and did not strongly correlate among each other, were included into the first regression model. Goodness of fit test is presented with chi-square, degrees of freedom, and significance value. The Cox & Snell R square and Negelkerke R Square values provide an indication of the amount of variation in the dependent variable explained by the logistic regression model. The predictive value of each independent variable is presented through Odds Ratio (OR). OR above 1 represents a positive correlation, while < 1 represents a negative correlation between dependent and independent variable within the logistic regression analyses. For the rest of the analyses, data are presented as mean(sd) and number of observants (%), and effect sizes for group differences by Hedges’ *g* (small: 0.2, medium: 0.5, large: 0.8) and Phi-coefficient (*φ*) (small: 0.1, medium: 0.3, large: 0.5) for numerical and categorical data, respectively. Alpha level was set to ≤ 0.05. Among all consenting students only one female exercise science student and one female reference student did not fully complete all questions. Therefore, no dropout differences were investigated.

**Table 1:** Participant characteristics presented as means (M) and standard deviation (sd) or total numbers (%)

	**Males**		**Females**		*P*	*g/φ*
	*N*	M (sd)	*N*	M (sd)		
Age	335	24.60 (4.83)	666	24.02 (4.71)	.068	
BMI (kg × m^2^)	334	24.55 (3.31)	661	24.00 (4.32)	**.024**	0.14
Immigration status	334	32 (9.6%)	666	63 (9.5%)	.951	
Study program						
Exercise science	333	220 (66.1%)	660	297 (45.0%)	**< .001**	0.20
Other sciences	333	113 (33.9%)	660	363 (55.0%)	**< .001**	0.20
PA, h/wk	307	9.18 (6.03)	588	7.19 (5.83)	**< .001**	0.34
Exercise sessions/wk	307	6.00 (2.80)	589	5.23 (2.59)	**< .001**	0.29
Organized sports members	307	137 (44.6%)	587	185 (31.5%)	**< .001**	0.13
Fitness center members	307	217 (70.7%)	587	438 (74.6%)	.207	
SATAQ-Athletic^*^	311	3.12 (0.96)	599	3.06 (0.92)	-	
SATAQ-Thin^*^	311	2.25 (0.98)	599	4.08 (0.61)	-	
SATAQ-General^*^	311	3.41 (0.96)	599	2.87 (1.03)	-	

Age: years of age. BMI: body mass index (kg/m^2^). SATAQ-4R: Social attitudes towards appearance questionnaire-4 revised. *SATAQ-4R includes male and female specific items, and gender differences are therefore not evaluated. PA h/wk: physical activity hours per week. Immigration status: the student and/or both parents have immigrated. A p-value of ≤0.05 is set as statistically significant when comparing two groups. *g*: Hedges’ *g* represents the effect size for numerical data and φ: Phi-coefficient represents effect size for categorical data and are only presented where there is a significant group difference

**Table 2:** Frequency of dietary supplement use among students shown as total number (N) and percentage (%)

	**Males**	**Females**	*p*	*φ*
DS	130 (42.3%)	232 (39.5%)	.414	
Vitamins and/or minerals	79 (23.6%)	194 (29.1%)	.063	
MEDS	82 (24.5%)	68 (10.2%)	** < .001**	(0.19)
Protein supplements	64 (19.1%)	60 (9.0%)	** < .001**	(0.15)
Creatin	50 (15%)	16 (2.4%)	** < .001**	(0.24)
Amino acids	10 (3%)	9 (1.4%)	.074	
Sports products^a^	47 (14.0%)	51 (7.7%)	**.001**	(0.10)
Dieting aids	4 (1.2%)	8 (1.2%)	***	
Herbal products	6 (1.8%)	8 (1.2%)	***	
Illegal supplements^b^	1 (0.3%)	2 (0.3%)	***	

*DS* dietary supplements; *MEDS* muscle enhancing dietary supplements. ^a^Students were asked about their use of sports product beyond protein and creatine supplementation (e.g. sports drinks, pre-workout, energy boosters, bars). ^b^Among different suggested illegal supplements that students could report use of, one male student reported use of growth hormones, while of the two female students reporting use of illegal supplements, central stimuli (*n* = 2), fat burners that were considered illegal (*n* = 1), sedatives (*n* = 1), and narcotics (*n* = 1), were reported. A *p*-value of ≤ 0.05 is set as statistically significant when comparing two groups. *φ:* Phi-coefficient is only presented where there is a significant group difference. *** group difference has not been investigated due to small sample size

### Ethics

The study was conducted according to the World Medical Association Declaration of Helsinki. It was approved by the Norwegian Regional Committees for medical and health research ethics (No. 33532) and the Norwegian Centre for Research data (No. 978522). The students consented to participate by pressing "yes" to the question of consent in an informative email and were redirected to the online questionnaire which was developed through the web-based system SurveyXact 8.2 offered by Ramböll, Aarhus, Denmark.

## Results

### Demographics

Participant characteristics are presented in Table 1. Among the 5344 students who were informed about the study, a total of 1001 male (335) and female (666) students consented to participate, with variation of number of respondents between variables. The mean (sd) age and BMI of the total sample was 24.21 (4.76) years and 24.18 (4.02) kg/m^2^, respectively, and in total, 9.5% of all students reported immigration background.

### Frequency of dietary supplement use

Vitamin, mineral, and protein supplement use was more frequent compared to other types of supplements (Table 2). The use of MEDS and sports supplements were more frequent in male compared to female students (*p* =  < 0.001) (Table 2). Due to the low frequency of dieting aids use among males and females, associations with, and explanations for, use of such supplements were not investigated further.

### Reasons for dietary supplement use

**Fig. 1:**
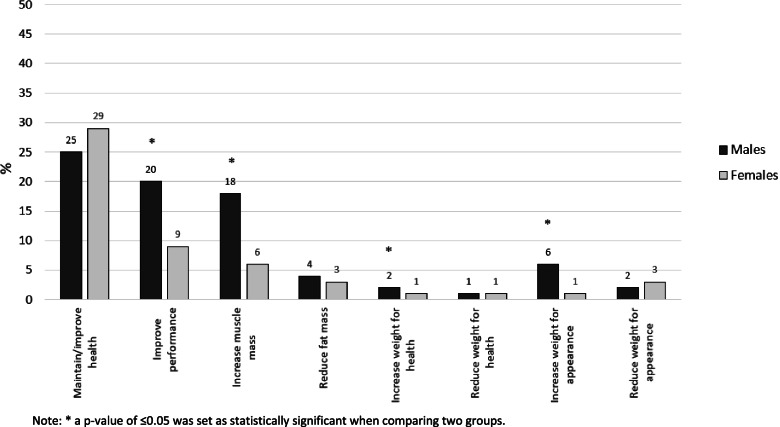
Differences in self-reported reasons for dietary supplement use between male and female university students

Among all response categories, “to maintain or improve health”, followed by “to improve mental or physical performance”, and “to increase muscle mass”, stood out as the most frequently reasons for use of DS among students regardless of gender (Fig. 1). More males than females reported to use supplements to improve physical or mental performance (*p* =  < 0.001, φ = 0.17), to increase muscle mass (*p* =  < 0.001, φ = 0.19), to increase weight for health (*p* = 0.014, φ = -0.08), and for appearance reasons (*p* =  < 0.001, φ = -0.17) (Fig. 1).

### Explanatory factors for use of muscle enhancing dietary supplements

#### Males

The correlational analyses (see additional Table 2) led to the inclusion of ten factors assumed to predict the use of MEDS. The full model containing all predictors was significant, *x*^2^ (10, *N* = 307) = 101.34, *p* =  < 0.001, and explained between 28.1% (Cox and Snell R square) and 40.9% (Nagelkerke R square) of the variance in MEDS use, and correctly classified 81.8% of the cases. Hours of physical activity per week, exercising to increase muscle mass, and being a fitness center member, made significantly positive contributions to the model, while exercising to increase endurance and being an exercise science student made statistical negative contributions to the model (Table 3). Being a fitness center member explained most of the use among male students, with an odds ratio of 3.81, after controlling for all other factors in the model.

**Table 3:** Logistic regression presenting likelihood of using muscle enhancing dietary supplements in male university students

	**B**	**S.E**	**Wald**	**df**	***p***	**OR**	**95% C.I. for OR**	
							Lower	Upper
Physical activity h/w	0.08	0.03	6.08	1	**.014**	1.09	1.02	1.16
Exercise sessions/w	0.03	0.08	0.11	1	.738	1.03	0.88	1.20
Exercise to increase muscle mass	0.68	0.26	6.69	1	**.010**	1.96	1.18	3.27
Exercise to improve strength	0.38	0.32	1.44	1	.231	1.47	0.78	2.74
Exercise to enhance endurance	-0.71	0.21	12.05	1	**.001**	0.49	0.33	0.73
Exercise to change body shape	0.12	0.17	1.41	1	.235	1.22	0.88	1.69
SATAQ Athletic	0.13	0.24	0.29	1	.588	1.14	0.71	1.84
Study program^a^	-0.76	0.35	4.58	1	**.032**	0.47	0.24	0.94
Fitness center member	1.34	0.50	7.19	1	**.007**	3.81	1.43	10.14
Participating in organized sport	0.39	0.38	1.02	1	.313	1.47	0.70	3.11
Constant	-5.541	1.430	15.019	1	.000	0.00		

*h/w* hours per week. ^a^Being an exercise science student or not. OR above 1 represents a positive correlation, while < 1 represents a negative correlation between dependent and independent variable

#### Females

The correlational analyses (see additional Table 2) led to the inclusion of eight factors assumed to predict the use of MEDS. The full model containing all predictors was significant, *x*^2^ (7, *N* = 587) = 46.66, *p* =  < 0.001, and explained between 7.6% (Cox and Snell R square) and 14.9% (Nagelkerke R square) of the variance in MEDS use, and correctly classified 88.4% of the cases. Exercising to increase muscle mass and internalization of the athletic body ideal made significant positive contributions to the model (Table 4). Internalization of the athletic body ideal explained most of the use among female students, with an odds ratio of 1.78, after controlling for all other factors in the model.

**Table 4:** Logistic regression presenting likelihood of using muscle enhancing dietary supplements in female university students

	**B**	**S.E**	**Wald**	**Df**	***p***	**OR**	**95% C.I. for OR**	
							Lower	Upper
Exercise sessions per week	0.06	0.06	0.91	1	.339	1.06	0.94	1.18
Exercise to increase muscle mass	0.55	0.26	4.40	1	**.036**	1.74	1.04	2.91
Exercise to improve strength	0.08	0.32	0.07	1	.795	1.09	0.58	2.07
Exercise to change body shape	0.11	0.12	0.82	1	.364	1.11	0.88	1.40
SATAQ Athletic	0.58	0.20	8.19	1	**.004**	1.78	1.20	2.64
Fitness center member	0.10	0.39	0.06	1	.801	1.10	0.52	2.35
Study program^a^	-0.13	0.30	0.20	1	.656	0.88	0.49	1.57
Constant	-7.39	1.31	31.69	1	.000	0.00		

^a^Being an exercise science student or not. OR above 1 represents a positive correlation, while < 1 represents a negative correlation between dependent and independent variable

## Discussion

### Summary of findings

This study aimed to explore the frequency of, and reasons for, DS use, and the frequency of, and explanatory factors for use of MEDS specifically, in male and female university students.

Among Norwegian university students, a similar frequency of males (42.3%) and females (39.5%) reported use of DS. Most students reported to maintain health, improve physical or mental performance, and to increase muscle mass, as reasons for DS use in general. As many as 1 in 4 males and 1 in 10 females used MEDS, with a significant gender difference. In males, being a fitness center member, exercising to improve muscle mass, and a higher physical activity level, positively explained use of MEDS, while exercising to increase endurance and being an exercise science student, negatively explained use of MEDS. In females, internalizing the athletic body ideal and exercising to improve muscle mass positively explained use of MEDS.

### Frequency of dietary supplement and muscle enhancing dietary supplement use

The frequency of DS and MEDS use in our sample is lower than previously reported [2, 5, 7–9]. Differences in BMI seem not to be explaining these differences, as BMI in the current sample is comparable to previous student samples [2, 5, 13, 35]. The differences in use of DS in general, and MEDS in particular, when comparing to other studies, might have been influenced by how “use” was defined, in which our study asked about “current use”, while previous studies asked about “the last six months” [2, 7, 8] or “the past 12 months” [9]. Our results indicate a low frequency of use of illegal DS (0.3% in both genders), which is lower than previous findings among Norwegian males (2.9%) and females (1.0%) trying out for the military basic training [38].

The lack of gender difference in use of DS in general, echoes what most studies from other countries have found [5, 6, 11, 13, 14]. However, our study is among few which have investigated gender differences for specific supplements, such as MEDS, and strengthens the assumption that males are more likely to use MEDS than females [5, 8, 11]. This gender difference might reflect a variance in advertisement targeted at males and females [39]. It could also reflect a more prolonged period of muscular body idealization within the history of masculinity, resulting in a more established focus on supplement use to meet this body ideal among males compared to females [40].

### Reasons for dietary supplement use

The most frequent reported reasons for use of DS among our university students, where “to maintain or improve health”, “improve mental or physical performance”, and to “increase muscle mass”, similar to what other student samples have reported [2, 5, 9–11]. In contrast to previous studies, we also looked at gender differences and found that significantly more males compared to females reported use of DS to improve performance and muscle mass, and to increase weight for appearance. This gender difference might underline the under-communicated body appearance pressure among male students [21], and their use of body modification methods.

### Explanatory factors for use of muscle enhancing dietary supplements

Among males, being a fitness center member explained most of the variance in MEDS use, indicated by being four times more likely to use MEDS compared to not being a member. This result is in line with findings among Norwegian high school students [24] but contributes with new in-depth information with regards to adults [31, 32]. Also, in line with findings among high school students, although a higher physical activity level slightly increased the odds of using MEDS in males, our findings are of no coherence between use of MEDS and participation in organized sports [24]. Possible explanations might be the culture within fitness centers, where sale and use of such products are promoted [31], and where a focus on body appearance and shaping dominates, as in contrast to the performance, enjoyment, health, and social focus, more prominently promoted within organized sports [29, 30, 41].

In males, reporting to exercise “to increase muscle mass” almost doubled the odds of using MEDS, which supports previous findings among male American fitness center members [32]. This might be expected, as those who aim to enhance muscle strength might be more receptive to advertisement claiming to give you a quick fix to improve muscle strength. In contrast, reporting to exercise “to increase endurance” reduced the odds of using MEDS, which is logical based on less intensive statements related to the products’ effects on endurance, and the lower need of muscle building when aiming to improve endurance.

Finally, among males, we also found that being an exercise science student, as compared to other students, reduced the odds (OR: 0.47) of using MEDS. Our finding adds to current knowledge about differences in MEDS use between student groups. Firstly, because previous studies investigating differences between study programs have investigated differences in DS use in general, not specifically MEDS. Secondly, previous studies included nurse, medicine, and pharmaceutical students, as health-related students, who in contrast to exercise science students, do not have an educational background in sports nutrition and exercise physiology, and represent a very different health-related student group. The education of exercise science students provides them with sports nutrition and exercise knowledge which might have equipped them with lifestyle related media literacy. This argument only relies on the authors knowledge about exercise science students’ curriculum, as we did not measure knowledge about supplements in our sample. However, one study supports our suggested explanation, by finding that students with higher media literacy were less likely to be DS users [14]. Also, exercise science students are exposed to a social environment which does not emphasize the need for supplements and special product in addition to, or at the expense of, real foods.

In female students, internalization of the athletic body ideal and exercising to increase muscle mass, being two highly coinciding elements, partly explained the variance in use of MEDS, after adjusting for all other variables in the regression model. The finding that females who internalized the athletic body ideal and exercised to increase muscle mass, were almost two times more likely to use MEDS, is novel among adults. However, it supports previous findings among Norwegian adolescent girls [24], and further underline that being occupied with improving physical appearance towards the muscular body ideal, takes part in explaining the use of MEDS.

Based on our finding that explanatory factors vary between genders, one could speculate whether males’ habits related to use of MEDS are more affected by the environment they engage in (exercise context and study program), while females’ habits are more strongly driven by their own demands and perceived needs to meet ideals (internalization). Importantly, in the discussion of explanatory factors for use of MEDS in both males and females, it is necessary to point out that the regression models for both genders only partly explained use of MEDS, which leaves room for further investigation of other explanatory factors which were not measured in this study. As an example, we were not able to assess energy and nutrient intake in the current study, and we only assessed current use of supplements without asking about duration and amount of use per supplement. The inclusion of these aspects of use might be especially important in future studies aiming to investigate and reflect upon potential long-term health consequences of such use. Also, considering our study design, we were not able to explore causation. Therefore, we cannot be sure whether students who are introduced to e.g., an exercise science program or a fitness center, develop specific behaviors in terms of using or not using MEDS, or whether individuals with specific characteristics are more prone to engage in such environments, and at the same time use such supplements. Longitudinal studies to better investigate direction of associations are needed in future research.

Importantly, our sample is comparable to previous student samples regarding age, BMI, and gender distribution within the sample. However, our sample differ by representing both exercise science students in addition to non-health and non-exercise science students. Also, in contrast to most studies, our sample included several universities, representing students from all parts of Norway.

### Strengths and limitations

Our study included a large sample size, compared both male and female students, and included scarcely investigated explanatory factors for use of MEDS. Despite the large sample size, a low response rate may impair generalization, and well-known biases are related to self-report of physical activity and exercise. Finally, based on our cross-sectional study design, cause-effect cannot be discussed.

### Perspectives

Because higher education is assumed to lead to enhanced competence to practice source criticism and to make evidence-based lifestyle choices, the high frequency of DS and MEDS use among Norwegian university students, is surprising. Although we found less frequent DS and MEDS use compared to previous studies, the frequency of, reasons for, and explanatory factors for use, such as appearance, is of concern due to the potential negative physical and mental health effects. Variables related to body idealization are found to reduce well-being and mental health. Therefore, the use of MEDS can be related to a condition of less favorable mental health. Since studying exercise science seemed to result in lower frequency of MEDS use in our study, increased knowledge about health, exercise, and nutrition, might be important to prevent uncritical use of such supplements. Changes are also necessary within the fitness center industry, as this exercise context is known to promote idealization of specific body types in addition to promoting and selling MEDS.

## Conclusion

Use of DS in general, and MEDS specifically, is frequent among Norwegian university students, with a higher frequency of MEDS use among males compared to females. Students most frequently used DS “to maintain or improve health”, “to improve mental or physical performance”, or “to increase muscle mass”. In relation to use of MEDS, internalizing the athletic body ideal, being a fitness center member, study program, exercising to increase muscle mass and to improve endurance, and physical activity level, partly explained use of MEDS, with important differences between males and females.

## Supplementary Information

**Additional file 1: Additional Table 1.** Characteristics of, and differences between, male and female users and non-users of muscle enhancing dietary supplements.

**Additional file 2:** **Additional** **Table 2**. Associations with use of muscle enhancing dietary supplements.

## Data Availability

The datasets generated and analyzed during the current study are not publicly available due to further use of the datafile in upcoming studies but are available from the corresponding author on reasonable request.

## Abbreviations

DS: Dietary supplements
MEDS: Muscle enhancing dietary supplements
SATA-Q: Social attitudes towards appearance questionnaire
OR: Odds ratio
SD: Standards deviation
CI: Confidence interval
M: Mean
S.E.: Standard error
