# Supplementary material for: Explanations for use of dietary- and muscle enhancing dietary supplements among university students: a national cross-sectional study
Source: BMC Nutr. 2022 Feb 24;8:17. doi: 10.1186/s40795-022-00510-1 (PMC8867755; doi:10.1186/s40795-022-00510-1)
Supplement: Supplementary file 1 — Additional file 1: Additional Table 1. Characteristics of, and differences between, male and female users and non-users of muscle enhancing dietary supplements. [file 40795_2022_510_MOESM1_ESM.docx]

**Additional Table 1**

Characteristics of users and non-users of muscle enhancing dietary supplements.

**Table 1.** Characteristics differences between students using or not-using muscle enhancing dietary supplements.

|  | Users | | Non-users | | *p* | *g/φ* |
| --- | --- | --- | --- | --- | --- | --- |
|  | *N* | M (sd) | *N* | M (sd) |  |  |
| MALES |  |  |  |  |  |  |
| Age | 82 | 23.99 (4.18) | 253 | 24.80 (5.01) | .187 |  |
| BMI kg×m^2^ | 82 | 24.96 (2.33) | 252 | 24.42 (3.56) | .118 |  |
| Immigration status |  | 6 (7.3%) |  | 26 (10.3%) | .423 |  |
| Study program (exercise science) |  | 63 (77%) |  | 157 (63%) | **.018** | 0.13 |
| Organized sports members |  | 26 (32%) |  | 111 (49%) | **.006** | -0.16 |
| Fitness center members |  | 76 (93%) |  | 141 (63%) | **<.001** | 0.29 |
| PA, h/wk | 82 | 10.99 (6.73) | 225 | 8.52 (5.62) | **.004** |  |
| Exercise sessions/wk | 82 | 6.76 (2.42) | 225 | 5.72 (2.88) | **.002** |  |
| Ex. to reduce body weight |  | 2.55 (1.23) |  | 2.68 (1.33) | .421 |  |
| Ex. to maintain body weight |  | 3.41 (0.98) |  | 3.22 (1.22) | .157 |  |
| Ex. to increase muscle mass |  | 4.41 (0.79) |  | 3.50 (1.12) | **<.001** |  |
| Ex. to reduce fat percentage |  | 3.33 (1.24) |  | 3.11 (1.36) | .204 |  |
| Ex. to improve strength |  | 4.62 (0.54) |  | 4.09 (1.01) | **<.001** |  |
| Ex. to enhance endurance |  | 3.94 (1.01) |  | 4.36 (0.83) | **.001** |  |
| Ex. enhance performance in sports |  | 3.59 (1.37) |  | 3.92 (1.30) | .053 |  |
| Ex. to change body shape |  | 3.65 (1.145) |  | 3.03 (1.24) | **<.001** |  |
| *SATAQ-Athletic* | 82 | 3.66 (0.80) | 229 | 2.92 (0.93) | **<.001** |  |
| *SATAQ-Thin* | 82 | 2.01 (0.89) | 229 | 2.33 (0.99) | **.010** |  |
| *SATAQ-General* | 82 | 3.71 (0.96) | 229 | 3.30 (0.94) | **.001** |  |
| FEMALES |  |  |  |  |  |  |
| Age | 68 | 23.68 (3.47) | 598 | 24.06 (4.83) | .527 |  |
| BMI kg×m^2^ | 68 | 23.06 (2.74) | 593 | 24.10 (4.45) | **.007** |  |
| Immigration status (%) |  | 4 (6%) |  | 59 (10%) | .287 |  |
| Study program |  | 37 (54%) |  | 260 (44%) | .100 |  |
| Participate in organized sports |  | 16 (24%) |  | 169 (33%) | .132 |  |
| Fitness center members |  | 58 (85%) |  | 380 (73%) | **.031** | 0.09 |
| PA, h/wk | 68 | 7.92 (4.43) | 520 | 7.09 (5.99) | .268 |  |
| Exercise sessions/wk | 68 | 6.01 (2.23) | 521 | 5.12 (2.62) | **.008** |  |
| Ex. to reduce body weight | 68 | 3.00 (1.45) | 521 | 3.34 (1.38) | .059 |  |
| Ex. to maintain body weight | 68 | 3.54 (1.09) | 521 | 3.53 (1.18) | .904 |  |
| Ex. to increase muscle mass | 68 | 4.53 (0.84) | 521 | 3.88 (1.02) | **<.001** |  |
| Ex. to reduce fat percentage | 68 | 3.78 (1.33) | 521 | 3.54 (1.29) | .156 |  |
| Ex. to improve strength | 68 | 4.78 (0.62) | 521 | 4.44 (0.84) | **<.001** |  |
| Ex. to enhance endurance | 68 | 4.57 (0.80) | 521 | 4.51 (0.75) | .493 |  |
| Ex. enhance performance in sports | 68 | 3.51 (1.47) | 521 | 3.48 (1.42) | .866 |  |
| Ex. to change body shape | 68 | 3.69 (1.23) | 521 | 3.30 (1.30) | **.020** |  |
| *SATAQ-Athletic* | 68 | 3.68 (0.80) | 531 | 2.98 (0.91) | **<.001** |  |
| *SATAQ-Thin* | 68 | 4.22 (0.60) | 531 | 4.06 (0.61) | **.040** |  |
| *SATAQ-General* | 68 | 2.85 (1.07) | 531 | 2.87 (1.03) | .887 |  |

*Note:* Table shows means and prevalence in male and female students Age: years of age. BMI: body mass index (kg/m^2^). SATAQ-4R: Social attitudes towards appearance questionnaire-4 revised with male and female specific items. PA h/wk: physical activity hours per week. Immigration status: the student and/or both parents have immigrated. g: Hedges’ *g* represents the effect size for numerical data and φ: Phi-coefficient represents effect size for categorical data. Effect sizes are only presented where there is a significant group difference. A *p*-value of ≤0.05 is set as statistically significant.
