# Supplementary material for: Explanations for use of dietary- and muscle enhancing dietary supplements among university students: a national cross-sectional study
Source: BMC Nutr. 2022 Feb 24;8:17. doi: 10.1186/s40795-022-00510-1 (PMC8867755; doi:10.1186/s40795-022-00510-1)
Supplement: Supplementary file 2 — Additional file 2: Additional Table 2. Associations with use of muscle enhancing dietary supplements. [file 40795_2022_510_MOESM2_ESM.docx]

**Additional Table 2**

Associations with use of muscle enhancing dietary supplements.

**Table 2.** Correlations with use of muscle enhancing dietary supplement in university students.

|  | Use of muscle enhancing dietary supplements | | | |
| --- | --- | --- | --- | --- |
|  | Males |  | Females |  |
|  | *r/φ** | *p* | *r/φ** | *p* |
| Age | -0.07 | .230 | -0.02 | .587 |
| BMI | 0.08 | .146 | -0.07 | .105 |
| Study program | 0.10* | .117 | 0.05* | .242 |
| PA h/week | 0.18 | **.001** | 0.05 | .268 |
| Exercise sessions/week | 0.16 | **.004** | 0.11 | **.008** |
| Fitness center member | 0.29* | **<.001** | 0.09* | **.045** |
| Participate in organized sports | -0.16* | **.009** | -0.06* | .171 |
| Exercise to reduce body weight | -0.05 | .421 | -0.08 | .061 |
| Exercise to maintain body weight | 0.07 | .199 | -0.01 | .889 |
| Exercise to increase muscle mass | 0.36 | **<.001** | 0.20 | **<.001** |
| Exercise to reduce fat percentage | 0.07 | .204 | 0.06 | .151 |
| Exercise to improve strength | 0.25 | **<.001** | 0.13 | **.001** |
| Exercise to enhance endurance | -0.21 | **<.001** | 0.03 | .487 |
| Exercise enhance performance in sports | -0.11 | .053 | 0.07 | .862 |
| Exercise to change body shape | 0.22 | **<.001** | 0.10 | **.020** |
| SATAQ-4 Athletic | 0.34 | **<.001** | 0.24 | **<.001** |
| SATAQ-4 Thin | -0.14 | **.014** | -0.01 | .875 |
| SATAQ-4 General appearance | 0.18 | **.001** | 0.08 | **.044** |

*Note:* Pearson Chi-square correlation coefficient (*r*) Chi-square test with Phi-coefficient (*φ**) represents effect size for numerical and categorical data, respectively. A p-value of ≤0.05 is set as statistically significant.
